# Glucagon promotes net hepatic glycogen repletion following meal ingestion

**DOI:** 10.1172/jci.insight.201076

**Published:** 2026-03-03

**Authors:** Nidhi Kejriwal, David Bouslov, Cheyenne R. Castle, Riya S. Karve, Galina A. Arkharova, Ashot Sargsyan, Daniel J. Drucker, Guo-Fang Zhang, David A. D’Alessio, Jonathan E. Campbell, Megan E. Capozzi

**Affiliations:** 1 Division of Metabolism, Endocrinology, and Nutrition, Department of Medicine, University of Washington, Seattle, Washington, USA.; 2 Duke Molecular Physiology Institute, Duke University, Durham, North Carolina, USA.; 3 Department of Medicine, Lunenfeld-Tanenbaum Research Institute, Mt. Sinai Hospital, University of Toronto, Toronto, Ontario, Canada.; 4 Department of Medicine, Division of Endocrinology, and; 5 Department of Pharmacology and Cancer Biology, Duke University, Durham, North Carolina, USA.

**Keywords:** Endocrinology, Metabolism, Diabetes, Glucose metabolism, Insulin

## Abstract

Insulin and glucagon are described as having opposing actions on hepatic glycogen metabolism. However, here we showed that their coordinated action promoted glycogen turnover and meal glucose storage. In mice, pharmacological doses of insulin or glucagon failed to alter hepatic glycogen, but the combination produced a robust decrease in glycogen content. Additivity between insulin and glucagon was also seen with the activation of hepatic insulin signaling intermediates. This signaling pathway drove glycogen synthesis, suggesting concurrent actions on glycogen breakdown and repletion. A mixed nutrient meal, which stimulates an increase in both insulin and glucagon, enhanced the incorporation of dietary glucose into hepatic glycogen. This was much more pronounced than the effects of glucose alone, which only stimulated insulin secretion. These findings revealed that glucagon is required for efficient hepatic glucose storage when acting in concert with insulin. Coordinated insulin-glucagon signaling, thus, emerged as a critical mechanism for hepatic glycogen cycling, challenging the classical paradigm that these hormones work in opposition.

## Abstract 2

When insulin and glucagon rise together after a meal, they work together, not in opposition, to help the liver efficiently store and manage carbohydrates.

## Introduction

Prandial glucose metabolism depends on the coordinated interaction of ingested nutrients, islet hormone secretion and action, and the liver’s complex, multifaceted response. The liver is crucial to carbohydrate metabolism where, in the case of meal ingestion, it can store a considerable amount of carbohydrate as glycogen. Modulation of hepatic glycogen is critical for maintaining euglycemia across varying nutritional states (1, 2). However, in patients with diabetes, hepatic glycogen storage is greatly attenuated (3–6), perhaps exacerbating the variability in glycemia experienced by these patients.

Hepatic glycogen content is predominantly regulated by insulin and glucagon. In response to a meal, insulin increases hepatic glycogen synthesis through an mTORC1-dependent activation of glycogen synthase activity (7). Conversely, under fasting conditions, glucagon is elevated, stimulating glycogen phosphorylase activity and, thus, catalyzing the breakdown of stored glycogen to provide the requisite fuel to combat hypoglycemia (8). Insulin and glucagon levels and/or action are dysregulated in diabetes; in the case of type 1 diabetes (T1D), β cells are lost, while in T2D, systemic insulin sensitivity and insulin production is reduced. Conversely, glucagon is not appropriately suppressed in response to carbohydrate ingestion during diabetes (9–12), further contributing to hyperglycemia. The lack of insulin action combined with elevated glucagon action may lead to the overall reduction in glycogen storage during diabetes.

Insulin and glucagon are classically viewed as opposing hormones because they have opposite effects on hepatic glucose metabolism and are secreted in inverse patterns in response to glucose. We and others have shown that glucagon can potentiate insulin secretion in a glucose-dependent manner through actions at the glucagon-like peptide-1 receptor (GLP-1R) and glucagon receptor (GCGR) (13–17), demonstrating a local interaction of insulin and glucagon when nutrients are elevated. Indeed, several studies have repositioned the role of glucagon with a focus on its role in amino acid metabolism (18–21). Glucagon secretion is highly responsive to several gluconeogenic amino acids, and loss of glucagon production or action leads to hyperaminoacidemia, constituting a liver-α cell axis (18, 20, 21). Moreover, mixed nutrient feeding has long been known to increase both insulin and glucagon levels (22), and this is associated with improved glucose tolerance to a matched carbohydrate-only challenge (23). Thus, while the normal response to mixed-nutrient meal ingestion is a simultaneous elevation in insulin and glucagon, how the coordinated action of these hormones modulates hepatic glycogen is incompletely understood.

Further supporting the coordinated actions of insulin and glucagon, recent studies have demonstrated a unique signaling signature in the liver, resulting from combined insulin and glucagon action (24, 25). In both isolated hepatocytes and rodent livers, pharmacological glucagon action potentiated aspects of canonical insulin signaling; insulin-stimulated phosphorylation of AKT^S473^ was potentiated by glucagon, while insulin-stimulated phosphorylation of AKT^T308^ was unaffected by glucagon. This glucagon-mediated potentiation of insulin action was shown to be mediated by mTORC2 activity and functionally leads to greater glucose lowering than insulin action alone. Moreover, a study of insulin-induced hypoglycemia in dogs demonstrated greater glucagon-stimulated hepatic amino acid extraction, an effect not observed when glucagon was elevated in fasting conditions (26). These findings support the potential for insulin and glucagon to communicate a unique hepatic response when acting together. However, these studies were assessing pharmacological administration of hormones, so the understanding of insulin and glucagon interaction under physiological conditions remains incomplete.

In the present study, we use both pharmacological administration of hormones and physiologic stimulation with mixed nutrient meals to further elucidate the unique actions of glucagon and insulin communication in the liver. We show that insulin potentiates glycogen depletion in response to glucagon. Moreover, a mixed nutrient challenge matched for carbohydrate composition to a glucose challenge leads to greater glycogen storage from meal glucose than glucose alone, despite no difference in absolute glycogen levels. Using glucagon loss-of-function models, we demonstrate that glucagon plays a role in hepatic glycogen repletion, counter to our current models of hormone action in glycogen metabolism. These studies alter the framework for understanding glucagon in hepatic glycogen metabolism and support future efforts to understand how to best implement glucagon pharmacology in the treatment of diabetes.

## Results

**Figure 1:**
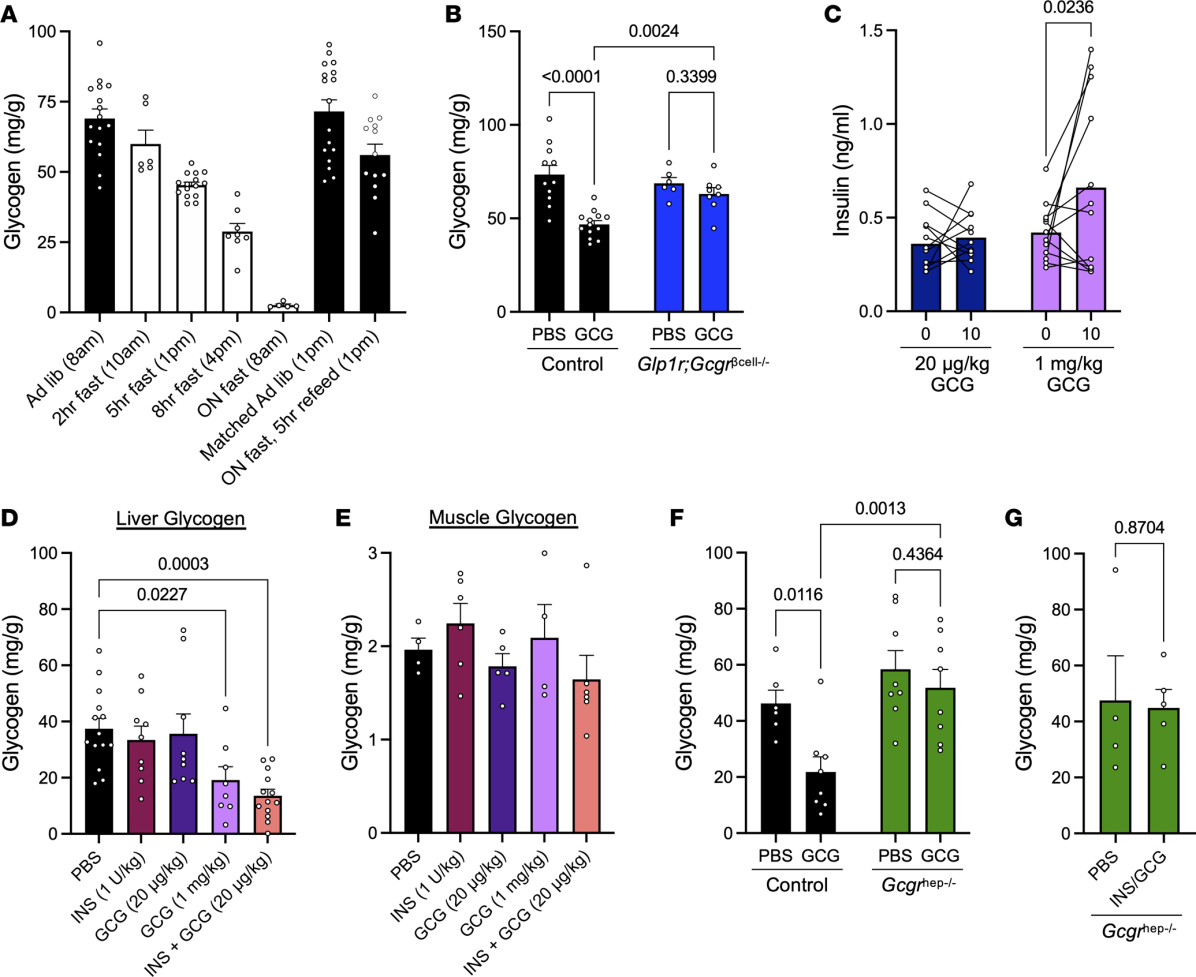
Glucagon and insulin interact to decrease hepatic glycogen. (**A**) Hepatic glycogen content in WT mice under various feeding (black bars) and fasting (white bars) conditions. (**B**) Liver glycogen 30 minutes following i.p. injection of PBS or glucagon (1 mg/kg) in Control and *Glp1r;Gcg*^rβcell–/–^ mice (*n* = 6–13 per group). (**C**) Plasma insulin levels at baseline and 10 minutes following 20 μg/kg glucagon or 1 mg/kg glucagon (*n* = 12 per group). (**D**) Liver glycogen 30 minutes after i.p. injection of PBS, insulin (1 U/kg), low-dose glucagon (20 μg/kg), high-dose glucagon (1 mg/kg), or insulin (1 U/kg) + low-dose glucagon (20 μg/kg) (*n* = 8–14 per group). (**E**) Glycogen in skeletal muscle 30 minutes after i.p. injection of PBS, insulin (1 U/kg), low-dose glucagon (20 μg/kg), high-dose glucagon (1 mg/kg), and insulin (1 U/kg) + low-dose glucagon (20 μg/kg). (**F**) Liver glycogen 30 minutes following i.p. injection of PBS or glucagon (1 mg/kg) in control and *Gcgr*^hep–/–^ mice (*n* = 6–8 per group). (**G**) Liver glycogen 30 minutes following i.p. injection of PBS and insulin (1 U/kg) + low-dose glucagon (20 μg/kg) in *Gcgr*^hep–/–^ mice (*n* = 4–5 per group). Data are shown as mean ± SEM. Two-way ANOVA (**B**, **C**, and **F**), 1-way ANOVA (**D** and **E**), or 2-tailed t-test (**G**) were used to determine significance, defined as *P* < 0.05.

We previously showed that high-dose glucagon (1 mg/kg) could ultimately lower blood glucose in a fed state in an insulin-dependent mechanism by acting to promote insulin secretion via both the GLP-1R and GCGR (13, 14). Based on the competing actions of insulin and glucagon to alter hepatic glycogen, we queried glycogen levels in the liver after hormone challenge. Mice were fasted overnight, followed by a 5-hour refeed to achieve comparably full, replete hepatic glycogen levels in all mice, as shown in Figure 1A. In response to i.p. injection of glucagon in the fed state, we observed a significant lowering of hepatic glycogen in WT mice, consistent with glucagon’s action to catalyze glycogenolysis (Figure 1B). Interestingly, when we challenged *Glp1r:Gcgr*^βcell–/–^ mice with glucagon, a model that lacks the insulinotropic actions of glucagon (14), hepatic glycogen levels remained unchanged (Figure 1B). This paradoxical finding suggests that glucagon’s interaction with insulin in the liver may potentiate glycogenolysis to a greater extent than glucagon alone.

To determine the extent to which insulin contributes to glucagon-driven hepatic glycogen depletion, we first performed pharmacologic studies in WT mice. We administered 2 glucagon doses to engage distinct receptor populations: a lower dose (20 μg/kg) to preferentially activate hepatic glucagon receptors and a higher dose (1 mg/kg) to additionally activate β cell GLP-1R and GCGR to stimulate insulin secretion (14), while still engaging hepatic GCGR. To contextualize the low-dose glucagon concentration and circulating levels achieved by i.p. injection over the time course of our studies, we measured glucagon concentrations in response to either 20 μg/kg glucagon or 2 U/kg insulin to show maximal endogenous glucagon production to hypoglycemia (Supplemental Figure 1A; supplemental material available online with this article; https://doi.org/10.1172/jci.insight.201076DS1). Additionally, to confirm the differential effects of these glucagon doses on islet insulin production, we showed that glucagon at 20 μg/kg did not stimulate insulin secretion, whereas 1 mg/kg glucagon significantly elevated circulating insulin levels (Figure 1C). Challenging WT mice fasted for 5 hours with either 1 U/kg insulin or 20 μg/kg glucagon had no effect on hepatic glycogen levels (Figure 1D), despite either intervention producing the anticipated effects on glycemia (Supplemental Figure 1B). After a 5-hour fast, we again observed lowering of hepatic glycogen in response to 1 mg/kg glucagon, consistent with increased glycogenolytic activity (Figure 1D). We hypothesized that the insulinotropic actions of the higher dose of glucagon were contributing to the effects on glycogen through the additive actions of hepatic glucagon and insulin signaling. To test this, we challenged mice with the combination of insulin (1 U/kg) and low-dose glucagon (20 μg/kg). Whereas the individual actions of either hormone did not affect glycogen levels, combining the 2 hormones produced a similar lowering of hepatic glycogen to that observed with high-dose glucagon (Figure 1D). Notably, the effect of exogenous hormone injection to lower glycogen was specific to the liver, as no effect was observed on muscle glycogen levels in any condition (Figure 1E), consistent with the lack of GCGR expression in muscle (27). Moreover, these effects were similar between male and female mice (Supplemental Figure 1C). Taken together, these findings show that the combined actions of pharmacological levels of insulin and/or glucagon lead to the greatest amount of hepatic glycogenolysis compared with the action of either hormone alone.

Due to the high concentrations of insulin and glucagon used for our initial studies, we sought to confirm that these effects were happening through direct action in the liver. We thus challenged mice lacking the hepatic glucagon receptor to determine whether the effects of glucagon and insulin were mediated directly through the liver or by another mechanism. *Gcgr^fl/fl^* mice were administered AAV8-TBG-CRE to induce hepatocyte-specific deletion (*Gcgr*^hep–/–^), with *Gcgr^fl/fl^* mice treated with AAV8-TBG-GFP serving as controls. As shown in Figure 1F, *Gcgr*^hep–/–^ mice had similar hepatic glycogen levels to controls after a 5-hour fast. However, when challenged with 1 mg/kg glucagon, glycogen levels remained unchanged compared with PBS-treated mice. Moreover, *Gcgr*^hep–/–^ mice did not lower hepatic glycogen content in response to insulin and glucagon–combined injection (Figure 1G). These data demonstrate that the observed effects of the combination of insulin and glucagon on hepatic glycogen are mediated through direct hormone action in the liver.

**Figure 2:**
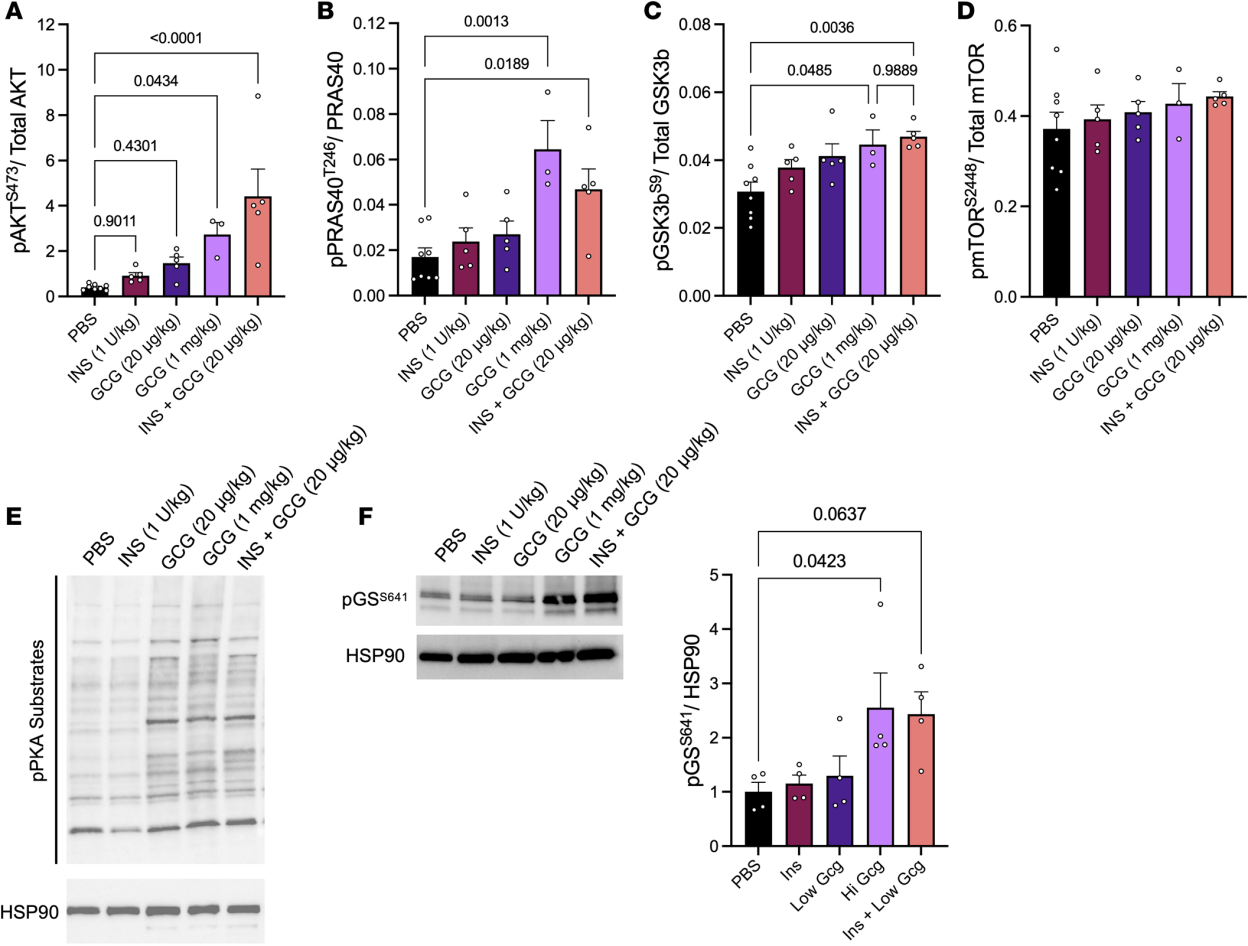
Insulin signaling is potentiated by glucagon action in the liver with no effect on glucagon action. (**A**–**D**) Insulin signaling intermediates (**A**) pAK^TS473^, (**B**) pPRAS4^0T246^, (**C**) pGSK3^bS9^, and (**D**) pmTO^RS2448^ from liver 10 minutes after i.p. injection of PBS, insulin (1 U/kg), low-dose glucagon (20 μg/kg), high-dose glucagon (1 mg/kg), and insulin + low-dose glucagon, quantified by Luminex-based Procarta Plex assay of (*n* = 5–8). (**E** and **F**) Representative Western blot of pPKA substrates (of 3 replicates) or pG^SS641^ from liver 10 minutes after i.p. injection of various treatments from above. HSP90 was used as the loading control (*n* = 3–4). Quantification of glycogen synthase phosphorylation is also shown in **F**. One-way ANOVA with Tukey’s post hoc was used to determine significance, defined as *P* < 0.05.

We next investigated canonical insulin and glucagon signaling to determine the extent to which insulin and glucagon may interact or compete to enact the changes observed in hepatic glycogen. After a 5-hour fast, WT mice were injected i.p. with insulin (1 U/kg), low-dose glucagon (20 μg/kg), high-dose glucagon (1 mg/kg), or combined insulin (1 U/kg) and low-dose glucagon (20 μg/kg). We first quantified phosphorylation of insulin-signaling intermediates of the AKT pathway, including AKT, PRAS40, GSK3b, and mTOR. We found that pAKT^S473^ and pPRAS40^T246^ were not significantly upregulated by insulin or low-dose glucagon (Figure 2, A and B). However, high-dose glucagon and the combination of insulin and low-dose glucagon stimulated pAKT^S473^ and pPRAS40^T246^. This corroborates findings that glucagon potentiates insulin action in the liver (24, 25). Similarly, we observed that pGSK3b^S9^ was significantly elevated by both high-dose glucagon and combined insulin and low-dose glucagon action, with no effect of low-dose glucagon or insulin alone (Figure 2C). Interestingly, phosphorylation at serine 9 inhibits GSK3b activity, which in turn promotes the activity of glycogen synthase (28), an enzyme that increases glycogen storage. However, in both treatment groups, glycogen levels are decreased (Figure 1D), suggesting that the combined actions of insulin and glucagon may promote concurrent glycogen repletion. We observed no difference with any treatment in pmTOR^S2448^ (Figure 2D), demonstrating that this target is likely not regulated by hormone action alone. We also assessed the effect of each treatment on hepatic glucagon receptor activity by Western blot for phosphorylation of PKA substrates, the primary signaling pathway engaged by GCGR activity. We found that glucagon increased pPKA substrates to a similar extent, whether in the presence or absence of stimulated insulin action (Figure 2E and Supplemental Figure 2). Lastly, we investigated the convergence of these pathways by quantifying phosphorylation of glycogen synthase. Phosphorylation at serine 641 done by protein phosphatase 1, and phosphorylation represents reduction of synthase activity. As shown in Figure 2F, increased glycogen synthase was observed in the 2 conditions where glycogen was most depleted, in response to high-dose glucagon or insulin + low-dose glucagon. These data demonstrate unique signaling engaged by the coordinated activity of insulin and glucagon when administered at pharmacological levels. Thus, we next investigated insulin and glucagon action in the physiologic state of meal ingestion.

**Figure 3:**
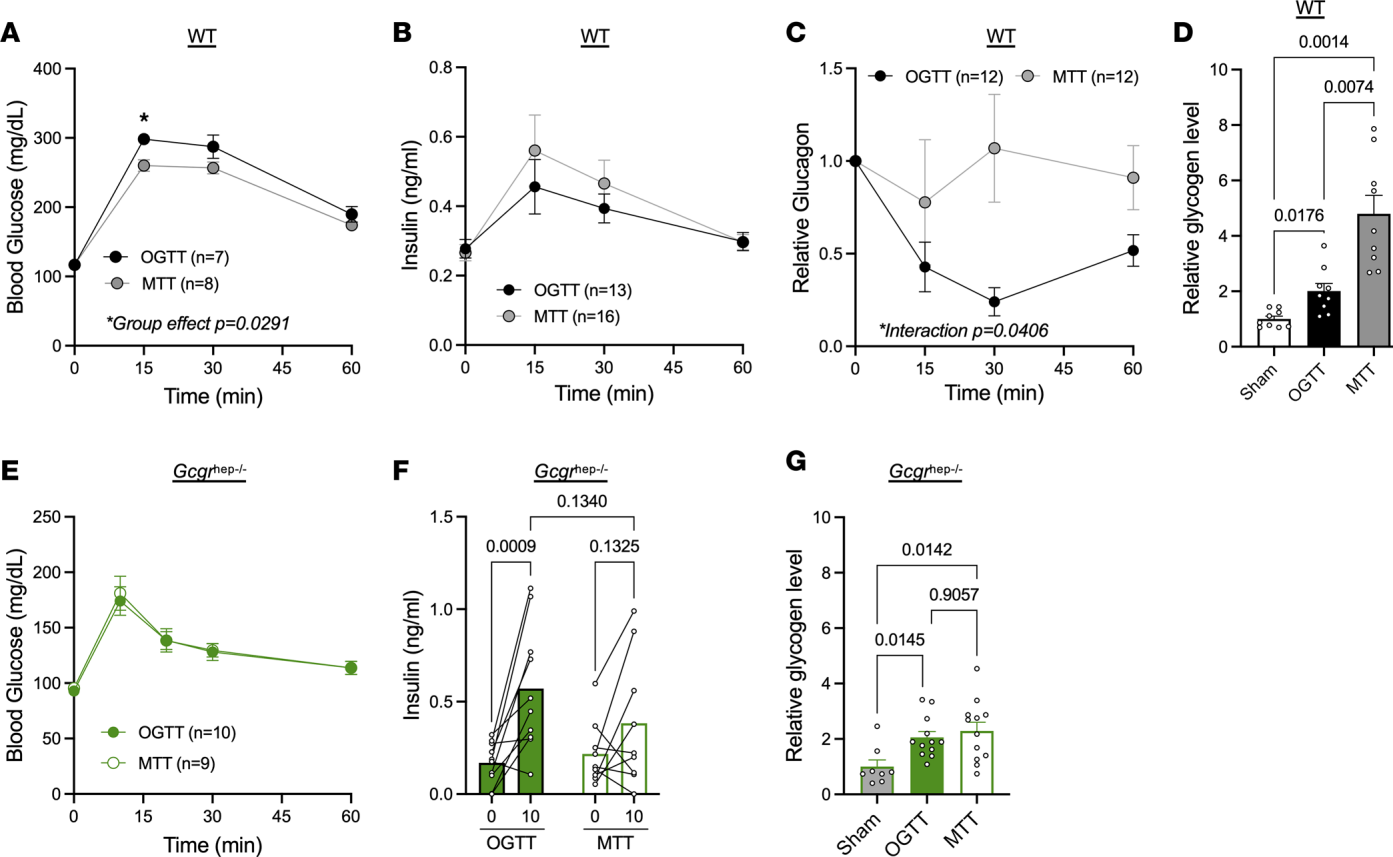
Hepatic glycogen repletion is improved after mixed nutrient feeding through hepatic glucagon action. (**A**–**F**) WT mice (**A–D**) or *Gcgr*^hep–/–^ mice (**E** and **F**) were fasted overnight before challenge of sham gavage control, glucose gavage (OGTT; 1.5 g/kg), or a mixed-nutrient meal matched for carbohydrate load (MTT; 10 μL/g Ensure). Blood glucose (**A** and **E**), insulin (**B** and **F**), and relative glucagon levels (**C**) were assessed at varying times within 60 minutes of gavage (*n* = 7–16). Relative glycogen content (**D** and **G**) was assessed 30 minutes after nutrient gavage (*n* = 8–10). Two-way ANOVA (**A–C**, **E**, and **F**) or 1-way ANOVA with Tukey’s post hoc (**D** and **G**) were used to determine significance, defined as *P* < 0.05.

As we previously demonstrated in WT mice, oral glucose stimulates insulin secretion with minimal effects on plasma glucagon, while a mixed-meal tolerant test (MTT) increases both insulin and glucagon plasma levels (23, 29). We confirmed these responses in mice challenged with either an oral glucose challenge (OGTT) or a MTT challenge (Ensure) matched for carbohydrate following an overnight fast, in which mice had nearly completely depleted hepatic glycogen levels (Figure 1A). Oral glucose resulted in a significantly greater glucose excursion compared with the carbohydrate-matched mixed-meal in WT mice (Figure 3A). While insulin responses did not differ between challenges (Figure 3B), glucagon levels were significantly suppressed only during the OGTT (Figure 3C). Consistent with our hypothesis that glucagon can potentiate insulin-induced glycogen accumulation, WT mice exhibited greater hepatic glycogen accumulation following MTT compared with OGTT, relative to sham-treated controls (Figure 3D).

To determine whether this enhanced hepatic glycogen repletion reflected the greater caloric content of the meal or was instead mediated by unsuppressed glucagon action in the liver, we compared OGTT and MTT responses in mice lacking the hepatic glucagon receptor (*Gcgr*^hep–/–^) mice. In these mice, OGTT and MTT resulted in similar glucose excursions (Figure 3E) and increased insulin levels (Figure 3F), although only OGTT significantly stimulated insulin secretion. While both challenges increased hepatic glycogen content to a degree comparable with WT mice, the additional hepatic glycogen accumulation following meal gavage was abolished in *Gcgr*^hep–/–^ mice (Figure 3G). Together, these findings indicate that intact hepatic glucagon action is required for normal meal-induced hepatic glycogen repletion, supporting a physiological role for glucagon beyond its classical function in promoting glycogen breakdown.

**Figure 4:**
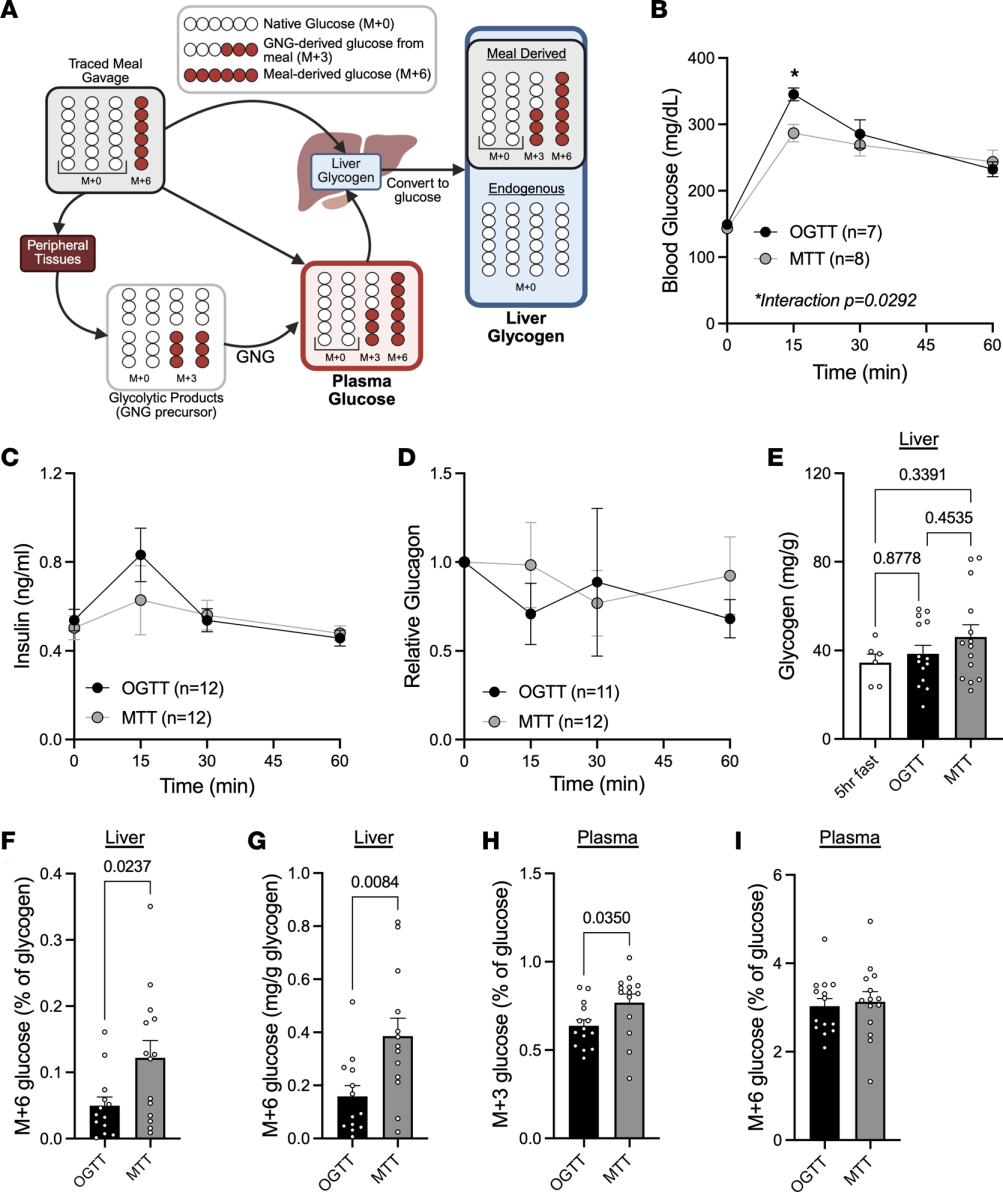
Mixed nutrient meal promotes greater meal glucose storage as glycogen than oral glucose matched for carbohydrate content. (**A**) Schematic of experimental strategy depicting source of glucose isotopologue (gluconeogenesis [GNG]). (**B**–**D**) Figure created with BioRender.com. Blood glucose (**B**), insulin (**C**), and relative glucagon levels (**D**) following oral gavage with oral glucose (OGTT; 1.5 g/kg; *n* = 7–12) or mixed-nutrient meal matched for carbohydrate load (MTT; 10 μL/g Ensure; *n* = 8–12). (**E**) Hepatic glycogen content after a 5-hour fast followed by sham gavage (*n* = 6), oral glucose (OGTT; 1.5g/kg; *n* = 14), or Ensure mixed meal (MTT; 10 uL/g; n = 14) in WT mice containing 10% ^13^C_6_-glucose. (**F**) Percentage of hepatic glycogen containing ^13^C_6_-glucose tracer (M+6 glucose) 60 minutes after gavage in OGTT and MTT (*n* = 14). (**G**) The estimated amount of meal glucose stored as hepatic glycogen after OGTT or MTT (*n* = 14). (**H**) Percentage of plasma M+3 glucose 60 minutes after gavage (*n* = 14). (**I**) Percentage of plasma M+6 glucose 60 minutes after gavage (*n* = 14). Two-way ANOVA (**B** and **C**), one-way ANOVA with Tukey’s post hoc (**E**) or 2-tailed t-test (**F–I**) were used to determine significance, defined as *P* < 0.05.

We next investigated how, under physiologic fasting conditions, meal-derived glucose is stored as glycogen and the role of glucagon in this process. To do so, we included ^13^C_6_-glucose in the OGTT and MTT to assess the fate of meal glucose as glycogen. The design of these experiments is depicted in Figure 4A; meal glucose is traced using M+6 glucose at 10% of the meal. The meal is gavaged and the recovery of that glucose is detected in plasma and liver glycogen, after conversion of glycogen to glucose. Detection of M+3 glucose reflects glucose derived from gluconeogenesis of glycolytic products, while M+6 glucose represents glucose derived from the meal and stored directly as glycogen or circulating without undergoing glycolysis at the time of collection. Consistent with prior observations (23, 30), OGTT resulted in a significantly greater glucose excursion than MTT (Figure 4B); however, circulating insulin and glucagon levels did not differ between the 2 interventions at the time points assessed (Figure 4, C and D). Notably, in a prior studies (14), we observed higher portal vein glucagon concentrations following MTT compared with OGTT, suggesting that peripheral plasma measurements may underestimate the differences in the hormonal milieu sensed by hepatocytes. Thus, despite similar systemic hormone levels, hepatic glucagon exposure during a mixed-nutrient meal may remain sufficient to influence intrahepatic glucose handling and glycogen synthesis.

We measured hepatic glycogen after a 5-hour fast, which leads to ~50% reduction in hepatic glycogen (Figure 1A) and allows for the possibility of glycogen turnover and repletion. Sixty minutes after either OGTT or MTT gavage, total hepatic glycogen did not increase compared with fasting conditions and was not significantly different between groups (Figure 4E). However, the percentage of glycogen derived from direct storage of meal glucose (termed M+6 glucose, derived from tracer signal) was greater following the MTT when compared with the OGTT (Figure 4F). To account for the inherent variability in the total glycogen measurement (Figure 4E), we quantified meal glucose stored as glycogen by calculating the percent of labeled glucose compared with the total glycogen measured (Figure 4G). We again observed a greater absolute amount of meal glucose stored as glycogen compared with oral glucose alone. No M+3 glucose was detected in hepatic glycogen (data not shown), demonstrating that the products of glucose breakdown, subsequent gluconeogenesis, and storage as glycogen were not important contributors to hepatic glycogen content. This was despite M+3 glucose being detectable in meaningful amounts in plasma, with greater levels following the MTT (Figure 4H). We observed no differences in circulating M+6 glucose between groups (Figure 4I). Overall, these data demonstrate that glucose from mixed-nutrient feeding is more efficiently stored as hepatic glycogen after gavage of carbohydrate-matched challenges.

We previously observed similar elevation of insulin levels between OGTT and MTT in WT mice (23). However, peripheral insulin levels may not represent activity in the liver, as there may also be differences in insulin clearance between the 2 meal challenges. We therefore assessed hepatic postreceptor insulin signaling in response to the various stimuli. Several canonical insulin signaling intermediates, including pAKT^S473^, pPRAS40^T246^, and pp70S6K^T421/S424^, were significantly increased to a similar degree following OGTT and MTT compared with sham-gavaged mice (Supplemental Figure 3, A–C), suggesting the differences in hepatic glucose storage as glycogen are not attributed to differences in insulin action. We did not observe a difference in pGSK3b^S9^ phosphorylation (Supplemental Figure 3D), but pmTOR^S2448^ was significantly elevated only following the MTT (Supplemental Figure 3E), suggesting that phosphorylation of mTOR at this site is regulated by nutrients as opposed to hormone action, as we did not observe an increase in mTOR phosphorylation with hormone injection (Figure 2D). Taken together, we showed that, in response to a mixed nutrient meal that is characterized by stimulation of both insulin and glucagon, meal glucose is more readily stored as glycogen than glucose challenge alone, and this is not due to differences in hepatic insulin action.

**Figure 5:**
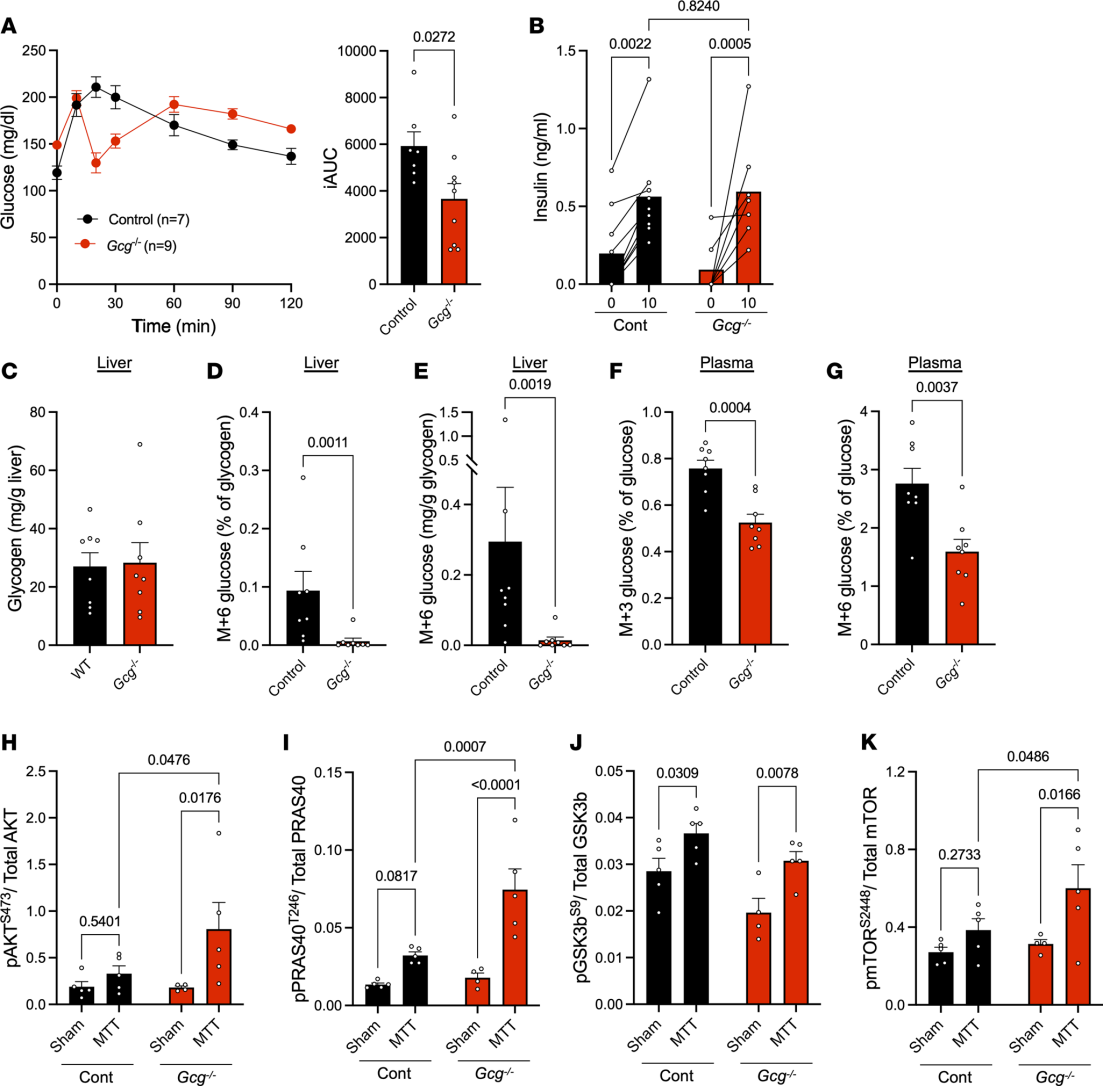
Proglucagon products are required for normal storage of meal glucose as hepatic glycogen. (**A**) Plasma glucose and integrated area under the curve (iAUC) from control (*n* = 7) and *Gc*g^–/–^ (n = 9) mice challenged with a mixed meal tolerance test (MTT; 10 μL/g Ensure). (**B**) Insulin levels at baseline and 10 minutes after MTT in control (n = 9) and *Gcg*^–/–^ (*n* = 7) mice. (**C**) Hepatic glycogen content after a 5-hour fast followed by gavage of a Ensure mixed meal (10 μL/g) containing 10% ^13^C_6_-glucose in control (*n* = 8) and *Gcg*^–/–^ (*n* = 8) mice. (**D**) Percent of hepatic glycogen containing ^13^C_6_-glucose tracer (M+6 glucose) 60 minutes after gavage in control and *Gcg*^–/–^ mice (*n* = 8). (**E**) The estimated amount of meal glucose stored as glycogen after mixed meal gavage in control and *Gcg*^–/–^ mice (*n* = 8). (**F**) Percentage of plasma M+3 glucose 60 minutes after gavage (*n* = 8). (**G**) Percentage of plasma M+6 glucose 60 minutes after gavage (*n* = 8). (**H**–**K**) Insulin signaling intermediates (**H**) pAKT^S473^ (**I**) pPRAS40^T246^ (**J**) pGSK3b^S9^ (**K**) pmTOR^S2448^ from liver 15 minutes after a sham or MTT gavage (*n* = 4-5), quantified by Luminex-based Procarta Plex assay. Two-tailed *t* test (**A** and **C–G**) or 2-way ANOVA (**B** and **H–K**) were used to determine significance, defined as *P* < 0.05.

To test the hypothesis that combined action of insulin and glucagon following a mixed nutrient meal stimulates more efficient glycogen storage, we performed meal challenges in 2 glucagon loss-of-function models. First, we studied mice lacking proglucagon products (*Gcg*^–/–^) (13, 31). *Gcg*^–/–^ mice had a significantly altered glycemic response to meal gavage, with overall improved glucose tolerance, as shown by a reduced integrated area under the curve (iAUC) compared with controls (Figure 5A), perhaps due to increased GIP sensitivity despite the loss of α cell to β cell communication as we have shown previously (13). Despite the dramatic glucose lowering observed between 10- and 20-minutes after meal gavage, meal-stimulated insulin was similar between control and *Gcg*^–/–^ mice (Figure 5B). Next, we performed meal tracer studies in these mice, using the experimental design shown in Figure 4A. Here, all mice received the same glucose tracer-labeled mixed nutrient meal. Sixty minutes after meal gavage, hepatic glycogen content was similar between WT and *Gcg*^–/–^ mice (Figure 5C), yet meal glucose storage as glycogen was nearly undetectable in *Gcg*^–/–^ mice and significantly reduced compared with cage-matched controls (Figure 5, D and E). While part of this decrease in meal glucose incorporation into hepatic glycogen could be attributed to decreased circulating levels of labeled plasma glucose (Figure 5, F and G), the decrement in circulating M+6 glucose was greater in liver glycogen than in plasma, suggesting additional mechanisms of altered glycogen storage. To confirm that the observed decreases in glycogen storage were not attributable to augmented insulin action in the liver, we assessed insulin signaling in the livers of control and *Gcg*^–/–^ mice 15 minutes after meal gavage challenge. Interestingly, we observed an overall trend of increased insulin action in *Gcg*^–/–^ mice compared with controls (Figure 5, H–K). Induction of both pAKT^S473^ and pPRAS40^T246^ by MTT was significantly greater in *Gcg*^–/–^ mice than in controls. Phosphorylation of GSK3b^S9^ was significantly elevated by MTT in the liver from both groups (Figure 5J). Lastly, we observed a greater stimulation of pmTOR^S2448^ by mixed nutrient gavage in *Gcg*^–/–^ than in controls (Figure 5K). This suggests that this phospho site is not responsible for increased mTORC1 activity that has previously been shown to be involved in catalyzing glycogen synthesis (7). These data show that proglucagon products are still important for meal glucose storage as glycogen, despite enhanced hepatic insulin action.

**Figure 6:**
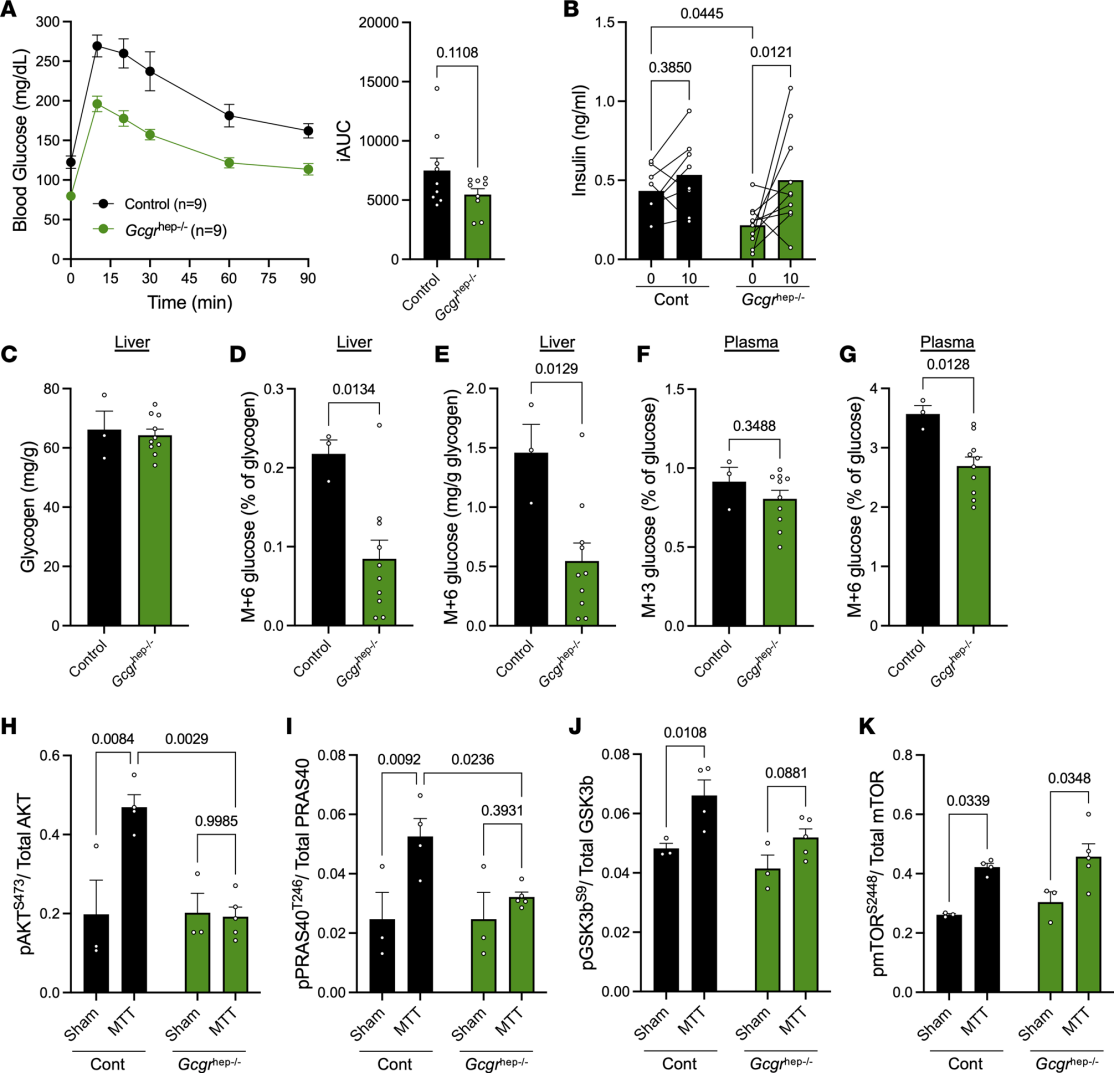
Hepatic glucagon action is required for normal storage of meal glucose as hepatic glycogen. (**A**) Plasma glucose and integrated area under the curve (iAUC) from control (*n* = 9) and *Gcgr*^hep–/–^ (*n* = 9) mice challenged with a mixed meal tolerance test (MTT; 10 μL/g Ensure) 1 week after AAV injection. (**B**) Insulin levels at baseline and 10 minutes after MTT gavage in control (*n* = 8) and *Gcgr*^hep–/–^ (*n* = 10) mice. (**C**) Hepatic glycogen content after a 5-hour fast followed by gavage of an Ensure mixed meal (10 μL/g) containing 10% ^13^C_6_-glucose in control (*n* = 3) and *Gcgr*^hep–/–^ (*n* = 10) mice. (**D**) Percent of hepatic glycogen containing ^13^C_6_-glucose tracer (M+6 glucose) 60 minutes after gavage in control (*n* = 3) and *Gcgr*^hep–/–^ (*n* = 10) mice. (**E**) The estimated amount of meal glucose stored as glycogen after MTT in control (*n* = 3) and *Gcgr*^hep–/–^ (*n* = 10) mice. (**F**) Percentage of plasma M+3 glucose 60 minutes after gavage. (**G**) Percentage of plasma M+6 glucose 60 minutes after gavage. (**H**–**K**) Insulin signaling intermediates (**H**) pAKT^S473^, (**I**) pPRAS40^T246^, (**J**) pGSK3b^S9^, and (**K**) pmTOR^S2448^ after a sham or MTT gavage (*n* = 3–5), quantified by Luminex-based Procarta Plex assay. Two-tailed *t* test (**A** and **C–G**), or 2-way ANOVA (**B** and **H–K**) were used to determine significance, defined as *P* < 0.05.

We lastly sought to use another approach to investigate the contribution of hepatic glucagon action to glycogen storage following a meal. We used AAV8-TBG-Cre injection to achieve hepatic glucagon receptor loss in a temporally controlled manner. Deletion of the hepatic glucagon receptor (*Gcgr*^hep–/–^) led to significant glucose lowering after a 5-hour fast (Figure 6A). While these mice maintained significantly reduced glucose over the course of an MTT, their overall glucose excursion was comparable with controls (Figure 6A). While *Gcgr*^hep–/–^ mice had lower baseline insulin levels than control mice, MTT increased insulin levels in these mice, similar to levels observed in controls (Figure 6B). We then assessed meal glucose stored as glycogen. Again, we observed similar hepatic glycogen content between control and *Gcgr*^hep–/–^ mice (Figure 6C). However, significantly less of the meal glucose was stored as glycogen in *Gcgr*^hep–/–^ mice compared with controls (Figure 6, D and E). In plasma, we saw no difference in M+3 glucose between *Gcgr*^hep–/–^ mice and controls, signifying no effect of hepatic glucagon activity for gluconeogenesis in the fed state (Figure 6F). However, these mice did have reduced circulating M+6 glucose (Figure 6G), which may partially contribute to reduced glucose storage as glycogen. While we observed only a ~25% decrease in M+6 glucose in plasma, we saw a ~65% decrease in meal glucose storage as glycogen.

Despite the MTT significantly elevating plasma insulin levels in *Gcgr*^hep–/–^ mice (Figure 6B), loss of the GCGR completely blunted meal-stimulated hepatic insulin action. While the meal effectively stimulated pAKT^S473^ and pPRAS40^T246^ in control mice, *Gcgr*^hep–/–^ mice had no meal-stimulated increase in either signal (Figure 6, H and I). Similarly, pGSK3b^S9^ was induced by MTT in controls but not in *Gcgr*^hep–/–^ mice (Figure 6J), suggesting decreased glycogen synthase activity in mice lacking the hepatic glucagon receptor. Phosphorylation of mTOR^S2448^ was elevated in both control and *Gcgr*^hep–/–^ mice (Figure 6K), further signifying that this pathway is regulated by meal nutrients rather than hormone activity alone and is not an indicator of glycogen synthesis activity.

## Discussion

**Figure 7:**
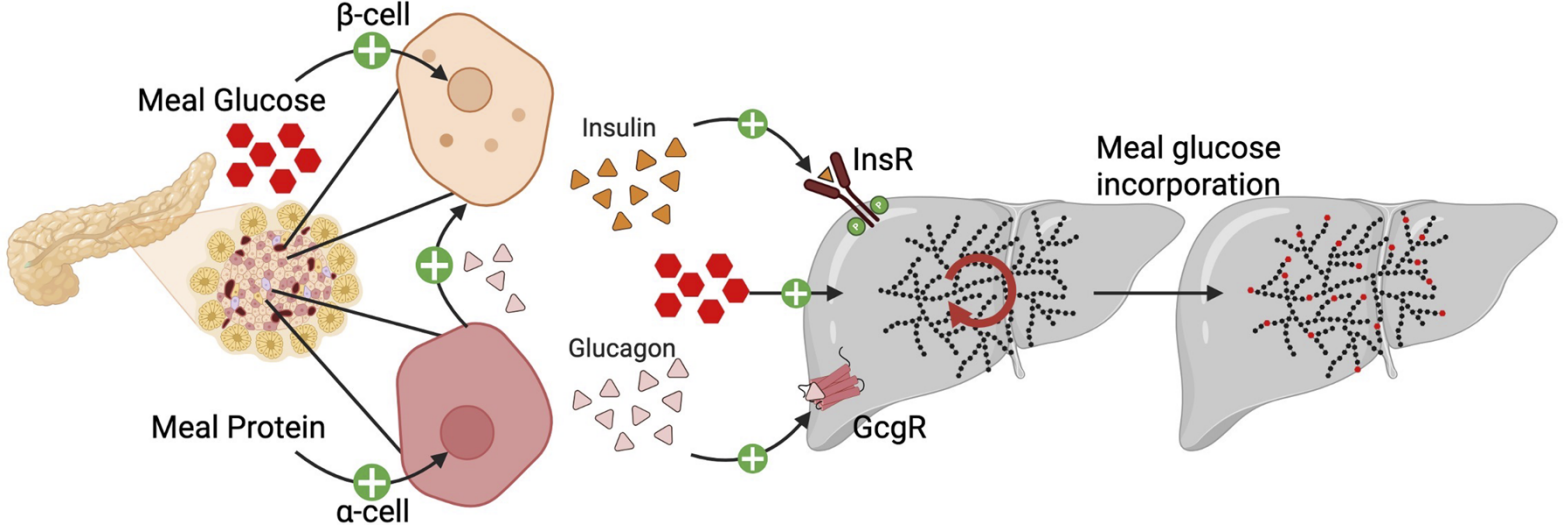
Glucagon works with insulin to store meal glucose as hepatic glycogen. An ingested meal activates both the β cell and α cell to secrete insulin and glucagon, respectively. Along with the meal macronutrients, insulin and glucagon enter portal circulation to communicate to the liver. The combined action of these hormones leads to simultaneous glycogen breakdown and repletion, leading to greater meal glucose storage as glycogen without a net overall increase in glycogen content. Figure created with BioRender.com.

We investigated how insulin and glucagon interact to regulate hepatic glycogen metabolism using a combination of pharmacologic hormone delivery, physiologic meal ingestion, genetic models of altered hormone interactions, and stable isotope tracer approaches in mice. A proposed model of our results is depicted in Figure 7. We found that, while pharmacological doses of either hormone alone are insufficient to alter hepatic glycogen levels, their combination produces robust glycogenolysis by coordinated activity that requires activation of the hepatic glucagon receptor. However, delivery of insulin and glucagon at pharmacological levels combined to induce a signaling profile typically associated with glycogen synthesis, suggesting that insulin and glucagon may promote concurrent glycogen breakdown and repletion.

Since our pharmacologic experiments lacked the elevation in circulating glucose substrate required for hepatic glycogen repletion in the presence of insulin (32, 33), we employed mixed-nutrient gavage to examine glycogen metabolism under physiologic feeding conditions. Using meal challenges and glucagon-deficient models, we observed that loss of glucagon signaling is associated with impaired incorporation of dietary glucose into hepatic glycogen following nutrient ingestion, despite intact or enhanced insulin signaling. We note that our experimental design prioritized preservation of endogenous insulin and glucagon temporal responses to nutrient intake rather than clamp-based approaches that impose fixed glycemic and hormonal conditions. Consequently, differences in glycemia in glucagon loss-of-function models limit direct attribution of these effects to glucagon action under matched glucose exposure. Within these constraints, our findings support a model in which glucagon contributes to efficient postprandial hepatic glycogen handling in concert with insulin.

Our findings reveal a previously underappreciated synergy between insulin and glucagon in the regulation of hepatic glycogen metabolism. The classical view holds that insulin and glucagon act in relative isolation, with insulin driving glycogen synthesis and glucagon promoting glycogenolysis. However, our data support that their coordinated action produces a unique hepatic response characterized by enhanced glycogen turnover and efficient nutrient storage after meals. These effects are liver specific, require an intact hepatic glucagon receptor, and are not attributable to changes in circulating glucose or insulin levels or hepatic insulin action alone. Interestingly, we did not observe reduced hepatic glycogen levels following low-dose glucagon treatment (Figure 1D), despite an increase in glycemia (Supplemental Figure 1B). This is likely due to the lack of sensitivity of this assay to detect small changes in glycogen, as previous studies using physiologic glucagon infusions in dogs, in the presence of basal insulin, demonstrated that glucagon-stimulated glucose output was predominantly due to hepatic glycogenolysis (34–36). However, our findings may also represent a species difference and/or difference in temporal hormone administration. We posit that the effects we observed may demonstrate a context-dependent partitioning of hepatic nutrient metabolism that is communicated in part by hepatic hormonal action. Future studies are needed to determine how hepatic nutrients are partitioned in response to different meal compositions or hormone inputs, as these factors are often dysregulated in diabetes and may contribute to disease pathogenesis.

In our previous work, we demonstrated that high-dose glucagon lowers blood glucose in an insulin-dependent manner, in part by stimulating insulin secretion via GLP-1 and GCGRs (14). Here, we build upon that observation by showing that glucagon’s ability to deplete hepatic glycogen similarly requires insulin action. Importantly, neither hormone at low dose alone induced significant hepatic glycogenolysis, but their combination did, suggesting a cooperative mechanism that surpasses the effect of either hormone in isolation. These results challenge the traditional binary model of glucagon and insulin as metabolic antagonists and, instead, support a model where their simultaneous action drives context-dependent metabolic outcomes.

Our mechanistic studies reinforce this interpretation. We observed that pharmacological doses of glucagon and insulin, alone or in combination, selectively modulate key hepatic signaling pathways. Notably, combined insulin and low-dose glucagon stimulated phosphorylation of AKT, PRAS40, and GSK3b, resembling the response to high-dose glucagon and suggesting that glucagon enhances hepatic insulin signaling under these conditions, an observation previously reported (24, 25). Interestingly, phosphorylation of GSK3b at serine 9, which is typically associated with activation of glycogen synthase and anabolic glycogen storage, was elevated in conditions where hepatic glycogen was instead depleted. This apparent paradox suggests that insulin and glucagon together may trigger dynamic glycogen cycling, characterized by concurrent synthesis and degradation. Such a mechanism would enable the liver to remain metabolically flexible, responding rapidly to shifting energy demands or nutrient flux. Indeed, glycogen flux has been reported in both rodents (37) and humans (38) in response to meal feeding.

To evaluate whether these interactions are relevant under physiological conditions, we compared hepatic glucose handling in mice subjected to oral glucose or mixed-meal tolerance tests (OGTT vs. MTT; Figures 3 and 4). Despite equivalent carbohydrate loads and similar insulin signaling activation, the MTT led to a significantly greater incorporation of meal-derived glucose into hepatic glycogen. This effect occurs with the physiological cosecretion of glucagon during mixed nutrient intake (23), implicating glucagon as a positive regulator of postprandial glycogen storage. The potential for glucagon to promote hepatic glycogen repletion was previously observed in exercise studies, where the presence or absence of glucagon with insulin did not affect overall hepatic glucose uptake; however, the conversion of glucose to glycogen was enhanced in the presence of glucagon (39), demonstrating a role for glucagon to affect glucose fate.

Loss-of-function studies further confirmed glucagon’s role in this process. Both global deletion of preproglucagon (*Gcg*^–/–^) and liver-specific deletion of the GCGR (*Gcgr*^hep–/–^) impaired the incorporation of labeled glucose into glycogen, and these effects were independent of the regulation of canonical insulin signaling intermediates. These data strongly argue that hepatic glucagon signaling is necessary for efficient glucose partitioning into glycogen following a meal, and that insulin action alone is insufficient. Notably, in *Gcgr*^hep–/–^ mice, postmeal insulin levels were comparable with controls, but downstream signaling (including pAKT, pPRAS40, and pGSK3b) was not activated, highlighting the importance of GCGR–mediated crosstalk for full hepatic insulin responsiveness. However, future studies are needed to delineate the contribution of altered glucose metabolism in glucagon loss-of-function models to altered glycogen metabolism.

Our studies do not support the idea that the improved glycogen storage is merely glucagon-potentiated insulin action that has been observed in other aspects of carbohydrate metabolism (24, 25). Indeed, when comparing OGTT with MTT in WT mice, which have differing circulating hormone profiles, stimulation of insulin signaling intermediates was similar between groups, while storage of glucose as glycogen was different (Figure 4). Moreover, we observed impaired glucose storage as glycogen in *Gcg*^–/–^ mice despite comparable, if not potentiated, insulin signaling in the liver (Figure 5). Understanding how glucagon may potentiate glycogen storage may be therapeutically useful, as hepatic glycogen storage is decreased in people with diabetes (3–6), and may lead to impaired hypoglycemic counterregulation (5, 40).

Our results may have relevance for the development of GCGR agonists and bihormonal closed-loop systems for diabetes management. Pharmacologic GCGR agonists are being explored for treatment of obesity and metabolic-associated steatotic liver disease (41). Traditionally, glucagon has been viewed as a catabolic hormone that raises blood glucose and, therefore, should be suppressed in diabetes (42). However, our data suggest that, under certain nutrient or hormonal conditions, glucagon action may facilitate hepatic glucose clearance and storage, especially when insulin is present. Indeed, the dual GLP-1R/GCGR agonist, cotadutide, is associated with increased hepatic glycogen content compared with vehicle control, GLP-1R monoagonism, or GCGR mono-agonism (43, 44), supporting the notion that combined insulin and glucagon can be useful for expanding hepatic glycogen in metabolic disease states. This context-dependent benefit of glucagon implies that selective or temporally controlled GCGR activation might be advantageous for restoring normal postprandial glucose handling in patients with metabolic disease.

Moreover, these findings may support improvements to bihormonal closed-loop insulin/glucagon delivery systems, which aim to better mimic physiological glucose control. Our data suggest that glucagon does not merely counteract insulin-induced hypoglycemia but rather can modulate insulin signaling and promote hepatic glucose disposal when delivered in concert. Incorporating glucagon into artificial pancreas systems could, therefore, support more physiologic hepatic glycogen cycling, improve meal-time glucose storage, and mitigate the risk of rebound hyperglycemia or hepatic insulin resistance. Indeed, recent studies have demonstrated reduced nutritional burden for patients after 1 year of a bihormonal closed-loop system (45).

While our data provide robust evidence of glucagon-insulin cooperation, they are based on pharmacological dosing and genetically modified mouse models. Whether these hormone interactions operate similarly in humans, particularly in insulin-resistant or diabetic states, remains to be determined. Future studies using stable isotope flux analyses, time-resolved tracer studies, or human liver models will be important for confirming translational relevance. It will also be important to evaluate how these mechanisms vary with nutritional state, metabolic disease, hepatic lipid content, and sex, all of which can influence hepatic metabolism.

In conclusion, we identify a synergistic interaction between insulin and glucagon that governs hepatic glycogen metabolism in a highly specific and dynamic manner. Far from opposing forces, these hormones can function cooperatively to regulate glycogen cycling, facilitate efficient nutrient storage, and optimize hepatic metabolic flexibility. This work challenges long-standing assumptions about glucagon’s role in metabolism and supports a model in which dual-hormone signaling promotes both glycogen breakdown and repletion. These insights may have implications for the design of metabolic therapies and artificial pancreas systems that more accurately reflect the integrated physiology of nutrient regulation.

## Methods

### Sex as a biological variable.

Males and females were used in all experiments. All data are grouped independently of sex, as there were no statistical differences between males and females in any endpoints tested.

### Animals.

Experiments were performed in 8- to 20-week-old mice on a C57BL/6J background. Mice had free access to a standard chow diet. WT mice were purchased from the Jackson Laboratory. *Glp1r;Gcgr*^βcell–/–^ mice were generated as described previously (13). Briefly, mice with *Glp1r*- and *Gcgr*-floxed alleles were crossed with *MIP-CreERT*^Cre/+^ to generate β cell–specific receptor deletion. *Glp1r*^fl/fl^;*Gcg*r^fl/fl^;*MIP-CreERT*^+/+^ served as controls. All mice were treated with 50 mg/kg tamoxifen in corn oil by oral gavage on 4 consecutive days at 6–8 weeks of age, and mice were used at least 1 month after tamoxifen treatment. *Gcg*^–/–^ mice were generated as described previously (13, 31). Heterozygous mice (*Gcg*^+/–^) were bred, producing *Gcg*^–/–^ mice and cage-matched *Gcg*^+/+^ controls. *Gcgr*-floxed mice were generated as described previously (46). Liver-specific KO was achieved by retro-orbital injection of AAV8-TBG-Cre, with floxed mice receiving AAV8-TBG-GFP injection serving as a control. These mice were used within 4 weeks after AAV injection.

### In vivo hormone and nutrient interventions.

For hormone challenges, mice were fasted for 5 hours and injected i.p. with insulin (1 U/kg), low-dose glucagon (20 μg/kg), high-dose glucagon (1 mg/kg), or combined insulin and low-dose glucagon. For nutrient challenges, mice were either fasted overnight or for 5 hours. Mice were then gavaged with glucose (OGTT; 1.5 g/kg) prepared in PBS or Ensure nutritional shake matched for carbohydrate load (MTT 10 μL/g). Ensure contains ~38 mg/mL protein, ~25 mg/mL fat, and ~135 mg/mL carbohydrate, representing ~15.7%, ~23.5%, and 55.7% of the total caloric value, respectively, approximating acceptable macronutrient distribution ranges (47). Blood glucose was measured using a glucometer (Contour Next). Plasma was collected using EDTA-coated capillary tubes, and insulin was measured by ELISA (Mercodia). For glucose labeling experiments, ^13^C_6_-glucose was added to the OGTT or MTT at 10% of the total glucose concentration. For tissue collection, mice were euthanized with CO_2_, and blood and tissue were collected in 15 minutes to 1 hour after intervention, as defined in each figure. In all experiments, injection volumes were 10 μL per gram body weight to control for the effect of volume.

### Glycogen quantification.

Frozen liver tissue (10–20 mg) was weighed on dry ice, incubated in 0.5N KOH at 95°C for 30 minutes with periodic vortexing, and then precipitated with 6% sodium sulfate and ethanol. The glycogen pellet was digested in 2 mg/mL amyloglucosidase in 0.2M sodium acetate (pH 4.9) at 50°C–55°C for 3 hours. Following dilution, 10 μL of each sample was mixed with 200 μL glucose oxidase reagent and incubated at 37°C for 10 minutes. Absorbance was measured at 500 nm, and glycogen content was determined using a standard curve generated from rabbit liver glycogen. Glycogen is expressed per tissue weight.

### Sample preparation and metabolite extraction from liver tissue.

Approximately 20 mg of liver tissue was placed into a prechilled Eppendorf tube and homogenized in 200 μL of methanol using a tissue homogenizer. Subsequently, 200 μL of distilled water and 200 μL of chloroform were added to the homogenate to initiate a modified Folch extraction. The mixture was vortexed thoroughly and centrifuged at 14,000*g* for 20 minutes at 4°C to achieve phase separation. The resulting upper aqueous phase (~350 μL) was carefully collected and evaporated to dryness under a gentle stream of nitrogen gas at 37°C. The dried extract was then reconstituted in 60 μL of distilled water, vortexed briefly, and transferred into an LC vial for subsequent liquid chromatography–mass spectrometry (LC-MS) analysis.

### LC-MS analysis for glucose labeling.

Glucose quantification was performed as previously described (48). Quantitative analysis was performed using a Vanquish LC system coupled to a Q Exactive Plus Orbitrap mass spectrometer (Thermo Fisher Scientific) operated in positive electrospray ionization mode. The chromatographic separation was carried out on a Microsorb-MV C18 column (100 × 4.6 mm, 3 μm) fitted with a matching C18 guard column. The column compartment was maintained at 40°C, and the autosampler was set to 5°C. A 1 μL aliquot of each sample was injected, and analytes were separated using isocratic elution with a mobile phase consisting of 98% H_2_O and 2% methanol containing 0.01% formic acid at a flow rate of 0.5 mL/minutes. The total run time per sample was 10 minutes.

The Q Exactive Plus mass spectrometer was equipped with a heated electrospray ionization (HESI) source. Ion source parameters were configured as follows: spray voltage at 3.5 kV, sheath gas at 30 arbitrary units, auxiliary gas at 13, sweep gas at 3, capillary temperature at 320°C, and heater temperature at 425°C. The S-lens RF level was set to 45. Full MS scans were acquired in the range of *m/z* 60–900 at a resolution of 70,000 (at *m/z* 200). The maximum injection time was set to 200 ms, with the automatic gain contro (AGC l) target set at 3 × 10^6^ ions.

### Western blot analysis.

Mouse liver tissues were homogenized in RIPA buffer containing protease inhibitors and phosphatase inhibitors (Cell Signaling Technology). Protein concentration of lysates was determined with a Pierce BCA assay (Thermo Fisher). Membranes were blocked in EveryBlot (Bio-Rad) and incubated in phospho-PKA substrate (Cell Signaling Technology; 9624), phospho-glycogen synthase (serine 641; Cell Signaling Technology; 4703), or HSP90 (Cell Signaling Technology; 4877). Bands were detected by the ChemiDoc Imaging System (Bio-Rad), and band density was quantified using the Bio-Rad Image Lab.

### ProcartaPlex AKT assay.

Mouse liver tissue was homogenized in ProcartaPlex Lysis Buffer (Thermo Fisher) containing PMSF and phosphatase inhibitors (Cell Signaling Technology). Protein was quantified using the Pierce BCA assay, and samples were prepared to 1 mg/mL concentration. Concentrations of phosphorylated (AKT^S473^; CREB^S133^; GSK-3b^S9^; IGF-1R^Y1135/Y1136^; IRS-1^S312^; mTOR^S2448^; PRAS40^T246^; p70S6K^T421/S424^), and respective total protein targets were assessed using the ProcartaPlex Akt Pathway 8-plex phospho and total panels (Thermo Fisher). The MAGPIX Luminex xMAP detection system was used to detect and quantify protein levels.

### Statistics.

Data are presented as mean ± SEM. Statistical analyses were performed using GraphPad Prism version 10. Comparisons between 2 groups were made using unpaired 2-tailed Student’s *t* tests. For experiments involving multiple groups, 1-way or 2-way ANOVA was used as appropriate, followed by Tukey’s post hoc test for multiple comparisons. *P* < 0.05 was considered statistically significant.

### Study approval.

All procedures were performed in accordance with approved protocols from either the Duke University or University of Washington IACUC.

### Data availability.

Values for all data points in graphs are reported in the Supporting Data Values file.

## Author contributions

Conceptualization was contributed by JEC and MEC. Methodology was contributed by GFZ and MEC. Investigation was contributed by NK, DB, CRC, RSK, GAA, AS, and MEC. Formal analysis was contributed by GFZ and MEC. Visualization was contributed by MEC. Resources were contributed by DJD, DAD, JEC, and MEC. Writing of the original draft was contributed by NK and MEC. Review and editing were contributed by NK, DB, CRC, RSK, GAA, AS, DJD, GFZ, DAD, JEC, and MEC. Funding acquisition was contributed by DAD, JEC, and MEC.

## Funding support

- Diabetes, Obesity and Metabolism Training Program (T32DK007247) and the University of Washington Diabetes Research Center (P30DK017047) (NK).
- Mary Gates Scholarship from the University of Washington (RSK).
- Banting and Best Diabetes Centre Novo Nordisk Chair in Incretin Biology, a Sinai Health Novo Nordisk Foundation Fund in Regulatory Peptides, and CIHR grant 154321 (DJD).
- Career development award from the NIH/NIDDK (K01DK129417) and a Pilot and Feasibility award from the University of Washington Diabetes Research Center (P30DK017047) (MEC.

## Conflict of interest

The authors have declared that no conflict of interest exists.

## Supplementary Material

Supplemental data

Unedited blot and gel images

Supporting data values

## Version 1 03/03/2026

In-Press Preview

## Version 2 04/22/2026

Electronic publication
