# Supplementary material for: Glucagon promotes net hepatic glycogen repletion following meal ingestion
Source: JCI Insight. 2026 Mar 3;11(8):e201076. doi: 10.1172/jci.insight.201076 (PMC13135395; doi:10.1172/jci.insight.201076)
Supplement: Supplemental data [file jciinsight-11-201076-s072.pdf]

Supplementary Figure 1

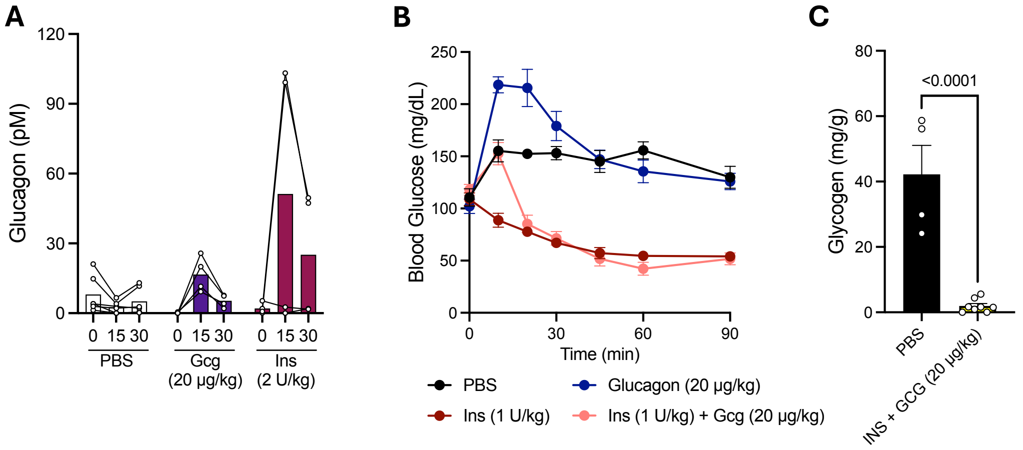

Supplementary Figure 1. (A) Circulating glucagon levels at baseline, 15 min, and 30 min following i.p. injection of PBS, glucagon (20 µg/kg), and insulin (2 U/kg). (B) Blood glucose levels after challenge with PBS, insulin (1 U/kg), glucagon (20 µg/kg), and insulin (1 U/kg) + glucagon (20 µg/kg). (C) Hepatic glycogen in WT female mice 30 minutes after i.p. injection of PBS or insulin (1 U/kg) + low-dose glucagon (20 µg/kg).

Supplementary Figure 2

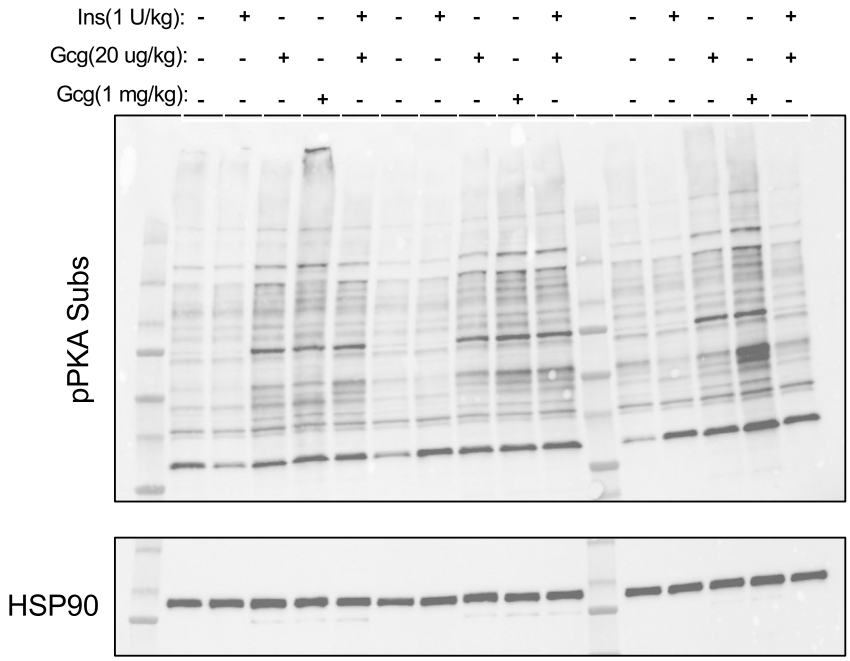

Supplementary Figure 2. Full, unedited western blot of pPKA substrates after ip injection of insulin (1 U/kg), low dose glucagon (20 μg/kg), high dose glucagon (1 mg/kg) or combined insulin and low dose glucagon. HSP90 was used as the loading control.

Supplementary Figure 3

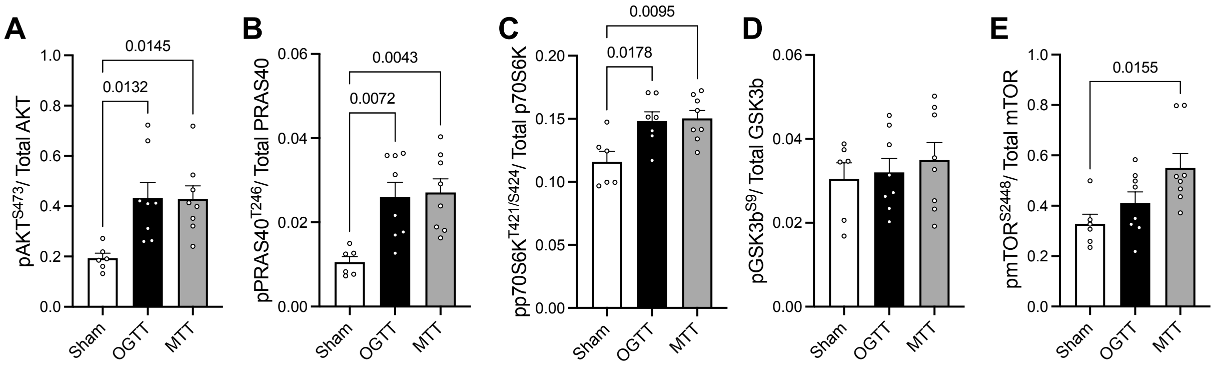

Supplementary Figure 3. Insulin signaling intermediates (F) $pAKT^{S473}$ (G) $pPRAS40^{T246}$ (H) $pp70S6K^{T421/S424}$ (I) $pGSK3b^{S9}$ (J) $pmTOR^{S2448}$ from liver 15 minutes after Sham (n = 6), OGTT (n = 8), or MTT gavage (n = 8), quantified by Luminex-based Procarta Plex assay. One-way ANOVA with Tukey's posthoc was used to determine significance, defined as $p<0.05$.
