# Supplementary material for: Glucagon promotes net hepatic glycogen repletion following meal ingestion
Source: JCI Insight. 2026 Mar 3;11(8):e201076. doi: 10.1172/jci.insight.201076 (PMC13135395; doi:10.1172/jci.insight.201076)

Unedited Blot from Figure 2E

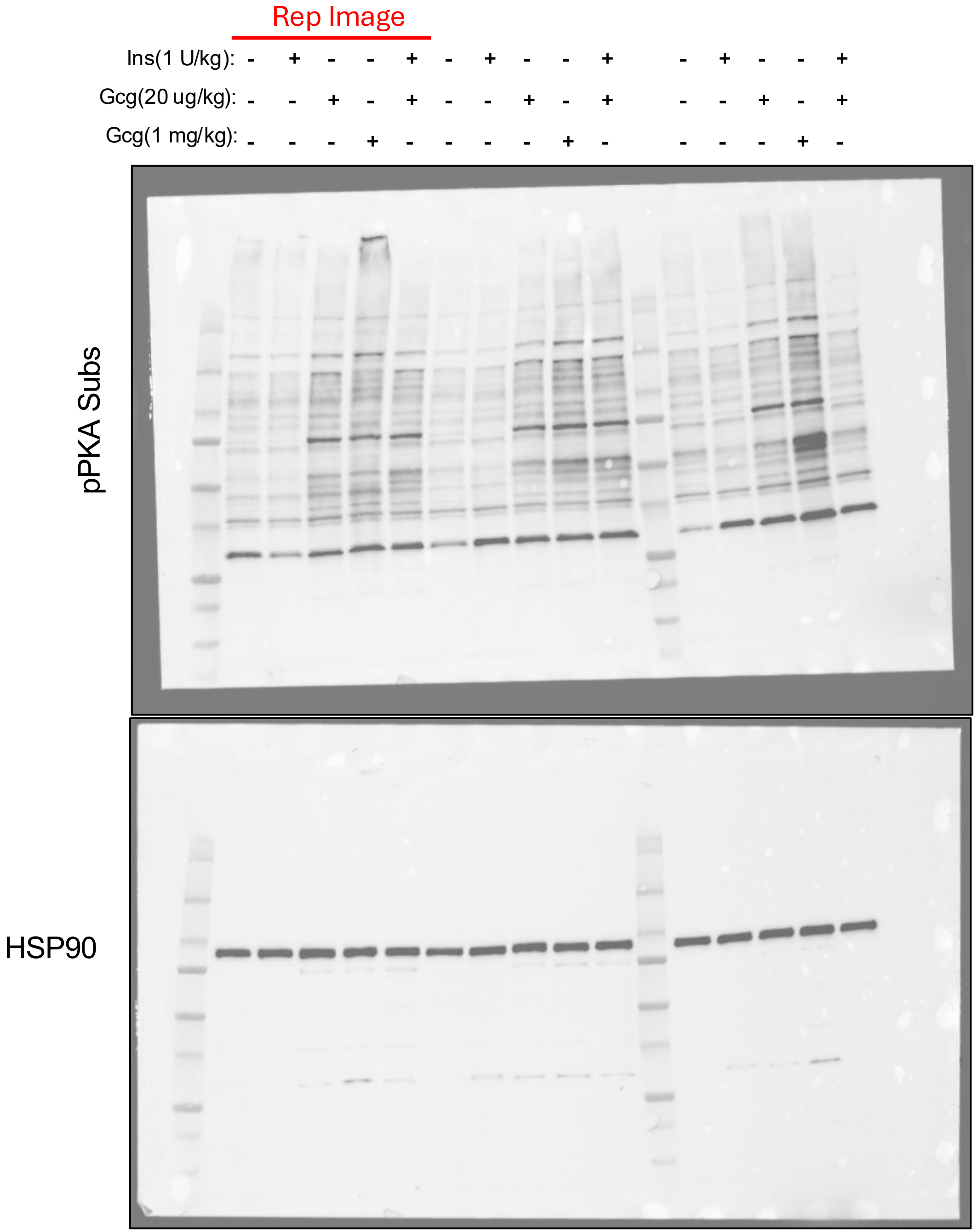

Unedited Blot from Figure 2F

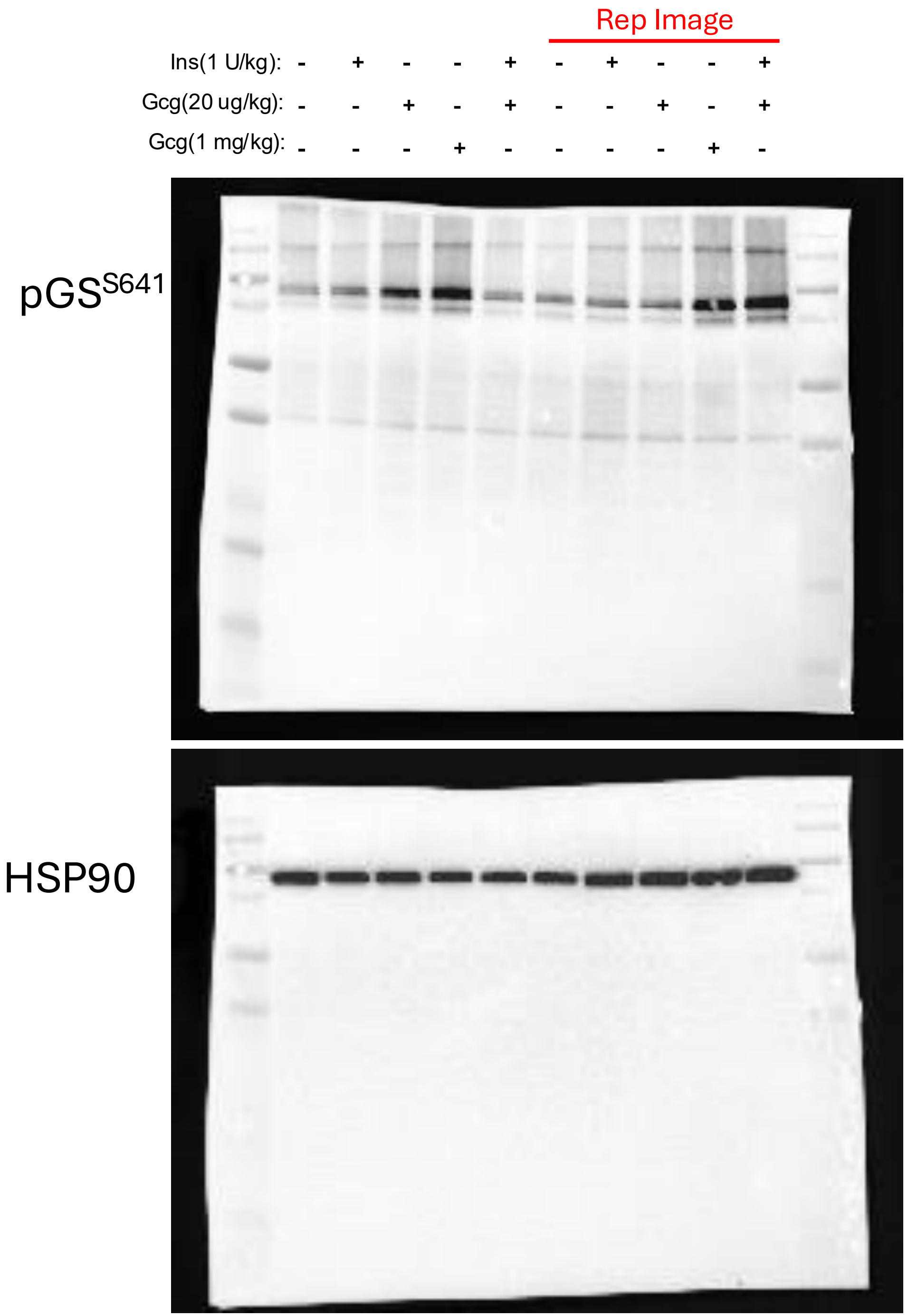

Supplement: Unedited blot and gel images [file jciinsight-11-201076-s073.pdf]
